# In vitro evaluation of chemical decontamination of titanium discs

**DOI:** 10.1038/s41598-021-02220-3

**Published:** 2021-11-23

**Authors:** Yuki Ichioka, Jan Derks, Gunnar Dahlén, Tord Berglundh, Lena Larsson

**Affiliations:** 1 Department of Periodontology, Institute of Odontology, Sahlgrenska Academy, University of Gothenburg, Box 450, 405 30 Gothenburg, Sweden; 2 Department of Oral Microbiology and Immunology, Institute of Odontology, Sahlgrenska Academy, University of Gothenburg, Gothenburg, Sweden

**Keywords:** Cell biology, Bacterial infection, Biomaterials - cells, Biomedical materials, Implants

## Abstract

Peri-implant diseases are caused by bacterial biofilm colonizing implant surfaces. Prevention and management of peri-implant mucositis and peri-implantitis rely on effective biofilm removal. This study aimed to evaluate biofilm removal and cytocompatibility following chemo-mechanical surface decontamination of biofilm-coated titanium discs. Biofilm-coated (*Streptococcus gordonii)* discs, with either non-modified (smooth) or modified (rough) surfaces, were instrumented using a sterile gauze soaked in one out of four solutions: saline (NaCl), alkaline electrized water (AEW), citric acid (CA) or *N*-acetyl-l-cysteine (NAC). Non-contaminated, untreated titanium discs served as controls (C). Residual deposits (bacteria and gauze fibers) and cytocompatibility for osteoblast-like cells were evaluated using SEM and immunofluorescence. Cytotoxicity was assessed using WST-8 assay and immunofluorescence. All protocols were equally effective in removing bacteria from smooth surfaces, while AEW and CA were found to be superior at rough surfaces. AEW and NAC were superior in promoting cytocompatibility over NaCl. NAC and CA had a strong cytotoxic effect on osteoblast-like and fibroblast cells. In conclusion, AEW may be beneficial in the decontamination of implant surfaces, effectively removing bacterial biofilm and restoring cytocompatibility.

## Introduction

Peri-implant diseases are inflammatory conditions driven by bacterial biofilm colonizing implant surfaces^1^. Both peri-implant mucositis and peri-implantitis are commonly observed biological complications in contemporary implant dentistry^2^. Mechanical instrumentation of implant surfaces, which may be non-modified (smooth) or modified (rough), is considered the cornerstone of treatment^3^, and chemical surface treatment has been suggested as an adjunctive tool in the management of peri-implant pathology^4,5^.

Mechanical instrumentation of implant surfaces requires proper access, which is frequently limited by the design of supra-constructions, by the configuration of the peri-implant bony defect and by the threaded design of the implant itself. Not only do chemical agents have the potential to clean otherwise non-accessible sites, they may also disrupt the chemical links between the underlying implant surface and the biofilm, critically damaging any biofilm structure. The use of chemical agents has been shown to critically reduce the biomass on contaminated titanium surfaces^6^. This anti-infective approach resulted in resolution of soft tissue inflammation and the absence of additional loss of supporting bone^4,7,8^. Both pre-clinical^9,10^and clinical studies^4,8^, however, clearly demonstrated that treatment outcomes were strongly dependent on surface characteristics.

It is understood that chemical surface properties are an important factor for cell/oxide layer interactions in the initial healing phase following implant instration^11–13^. Observations from *in-vitro* studies^14,15^ indicated that bacterial plaque contamination has the potential to alter the composition of the titanium oxide layer. Ichioka et al.^15^ demonstrated that even when biofilm was completely removed, chemical surface properties and cytocompatibility could not be restored to pristine levels. The understanding in regard to so called biofunctionalization of previously contaminated implant surfaces is limited.

The present study focuses on two chemical agents, i.e. alkaline electrolyzed water (AEW) and *N*-acetyl-l-cysteine (NAC). AEW contains a hypochlorite ion (^–^OCl) and hypochlorous acid (HOCl) and was reported to have antimicrobial effects and to remove organic impurities from hard material surfaces^16–18^. NAC, a derivative of the amino acid L-cysteine, is a potent thiol-containing antioxidant and mucolytic agent that disrupts disulfide bonds in mucus and reduces the viscosity of secretions^19,20^. NAC is widely used in medical practice via inhalation, oral and intravenous routes and has an excellent safety profile^21^.

*In-vitro* experiments may provide relevant findings in regard to cleaning potential of different chemical agents and studies applying biofilm-related experimental models commonly used single-bacteria biofilm^6,15,22–26^. *S. gordonii* is recognized as an initial colonizer of implant surfaces and as a component of the bacterial biofilm associated with peri-implantitis^27,28^. The aim of the present study was to evaluate the effect of the adjunctive use of chemical agents on biofilm removal and cytocompatibility of previously contaminated titanium discs. An additional aim was to assess cell viability and DNA damage in osteoblast-like cells (MG63) and human gingival fibroblasts (HGF).

## Materials and methods

### Titanium discs

Sterile titanium discs (Dentsply Sirona Implants, Mölndal, Sweden; diameter: 12 mm; thickness: 2 mm) with two different surface characteristics were used: 1. Non-modified surface [Ti(s)]. Surface topography measurements; Sa (arithmetical mean height): 0.187 ± 0.011 µm, Sdr (developed interfacial area ratio): 3.631 ± 0.283%, Sds (density of summits): 0.131 ± 0.007 summits/µm^2^, and 2. Modified (blasted using TiO_2_ particles) surface [Ti(r)]. Surface topography measurements; Sa: 1.546 ± 0.017 µm, Sdr: 66.711 ± 2.843%, Sds: 0.409 ± 0.002 summits/µm^2^. The surface roughness of the two categories of discs was evaluated using white light interferometry (MicroXAM, ADE Phase Shift Technology, USA). Non-contaminated and untreated titanium discs were used as pristine controls (C). All discs were cleaned according to standard protocols applied to implants for clinical use and autoclaved.

### Bacterial strain and culture conditions

*Streptococcus gordonii* ATCC 33399^ T^ (Culture Collection University of Gothenburg, Gothenburg, Sweden) was used in this study. Bacteria were cultured on horse blood agar plates (Clinical Microbiology, Sahlgrenska University Hospital, Gothenburg, Sweden) under aerobic conditions (N_2_: CO_2_/90%: 10%) at 37 °C for 3 days. Single colonies of *S. gordonii* were transferred from the house blood agar plates into Brain Heart Infusion liquid culture medium (Neogen, Lansing, MI, USA). Turbidity was calibrated by measuring the absorption at OD660: 0.5.

### Saliva preparation

Saliva was collected in a 50 ml tube from one of the investigators (Y.I) and heated at 60 °C for 30 min in a water bath to inactivate endogenous enzymes and then centrifuged (4500×*g*) for 30 min at 4 °C. The supernatant was diluted (1:1) with phosphate buffered saline (PBS). The saliva was passed through a 0.22 μm filter to remove microorganisms and stored at − 20 °C. To test the sterility of saliva, 30 μl were plated onto three house blood agar plates (Clinical Microbiology, Sahlgrenska University Hospital), which were subsequently cultured for 72 h at 37 °C under aerobic conditions (N_2_: CO_2_/90%: 10%). Saliva was considered to be sterile if no bacteria growth could be detected. The sterile saliva was used to coat titanium discs prior to biofilm development.

### Biofilm development on titanium discs

Titanium discs coated with saliva for 4 h at 37 °C were immersed in bacterial suspension (1.0 ml) using 24-well polystyrene culture plates. The plates were incubated for 48 h under aerobic conditions (N_2_: CO_2_/90%: 10%) at 37 °C for biofilm formation. Subsequently, all contaminated discs were gently washed twice with PBS and were then transferred into a new sterile 24-well plate for further experimental steps.

### Chemical surface decontamination procedures

Biofilm-contaminated titanium discs were cleaned using sterile gauzes (10 × 10 mm) soaked in (1) 0.9% saline (NaCl; pH 7.0, Fresenius Kabi, Sweden), (2) 0.1% alkaline electrized water (AEW; pH 9.0, Aoi Engineering Inc., Shizuoka, Japan), (3) 40% citric acid (CA; pH 1.0, citric acid monohydrate, Merck KGaA, Darmstadt, Germany) or (iv) 10% *N*-acetyl-l-cysteine (NAC; pH 1.0, Sigma-Aldrich, Sweden). The goal of the decontamination procedures for all four groups was complete removal of biofilm over the entire surface of the disc. Cleaning was carried out with a constant and overlapping linear movement for 60 s by one trained operator (YI). Gauzes were exchanged after 30 s. Following treatment, titanium discs were gently rinsed with 10 ml sterile saline to remove any potential deposits. Treated discs were then transferred into a new sterile 24-well plate for further analysis.

### Cell culturing

Osteoblast-like cells (human osteosarcoma cell; MG63; Sigma-Aldrich Sweden AB, Stockholm, Sweden) were cultured using Minimum Essential Medium Eagle (MEME; Sigma-Aldrich) supplemented with 10% newborn calf serum (NCS; Sigma-Aldrich), 1% of antibiotic–antimycotic (contains 100 units/ml penicillin, 100 μg/ml streptomycin, 250 ng/ml amphotericin B; Thermo Fisher Scientific), 1% of non-essential amino acids solution (Thermo Fisher Scientific) and 1% of GlutaMAX (contains L-alanyl-L-glutamine dipeptide; Thermo Fisher Scientific). Cells were incubated in a humidified atmosphere of 95% air and 5% carbon dioxide at 37 °C. The culture medium was replaced every 3 days. At approximately 80% confluency, cells were detached using 0.05% trypsin-0.53 mM EDTA-4 Na (Invitrogen, Ltd., Paisley, UK) at 37 °C for 5 min and, immediately after surface decontamination, seeded onto control and treated titanium discs in a 24-wells plate. Cells were cultured using MEME supplemented with 10% NCS without antibiotics.

Fibroblasts (primary human gingival fibroblast; HGF, ATCC® PCS-201–018™) were cultured using Fibroblast basal medium (ATCC® PCS-201-030™, LGC Standards, Wesel, Germany) and Fibroblast growth kit-low serum (ATCC® PCS-201-041™, LGC Standards) in a humidified atmosphere of 95% air and 5% carbon dioxide at 37 °C. HGF-cells were used for cytotoxicity testing.

### Scanning electron microscopy (SEM) analyses

SEM (GeminiSEM 450, ZEISS, Germany) was used to detect attached cells, residual bacteria and remnants of gauze fibers. Six titanium discs (3 Ti(s) & 3 Ti(r)) from each of the control and the 4 different treatment groups were placed into a 24-wells plate immediately after surface decontamination and MG63-cells were seeded at a density of 1.0 × 10^4^ cells/cm^2^. After 24 h incubation in a humidified atmosphere of 95% air and 5% carbon dioxide at 37 °C, discs were gently washed twice using PBS to remove unattached cells. Attached cells on titanium discs were fixed in 2.5% glutaraldehyde (TedPella, Redding, CA, USA) buffered in 0.1 M PIPEs buffer (Sigma-Aldrich) for 60 min. After fixation, discs were incubated in 1% osmium tetroxide (TAAB, Aldermaston, UK) in 0.1 M PIPEs buffer for 30 min in a cold and dark room. After washing with distilled water (5 × 3 min), discs were incubated in filtered 1% thiocarbohydrazide (EMS, Hatfield, PA) at room temperature for 10 min and washed with distilled water (5 × 3 min). Thereafter, they were incubated in 1% osmium tetroxide at 4 °C in the dark for 30 min and washed with distilled water (5 × 3 min). Discs were dehydrated in 30%, 50%, 70%, 85% and 95% ethanol (Fischer Scientific, Gothenburg, Sweden) for 5 min respectively and 100% ethanol for 20 min. Discs were finally dried using hexamethyldisilazane (Fluka, Buchs, Switzerland).

**Figure 1:**
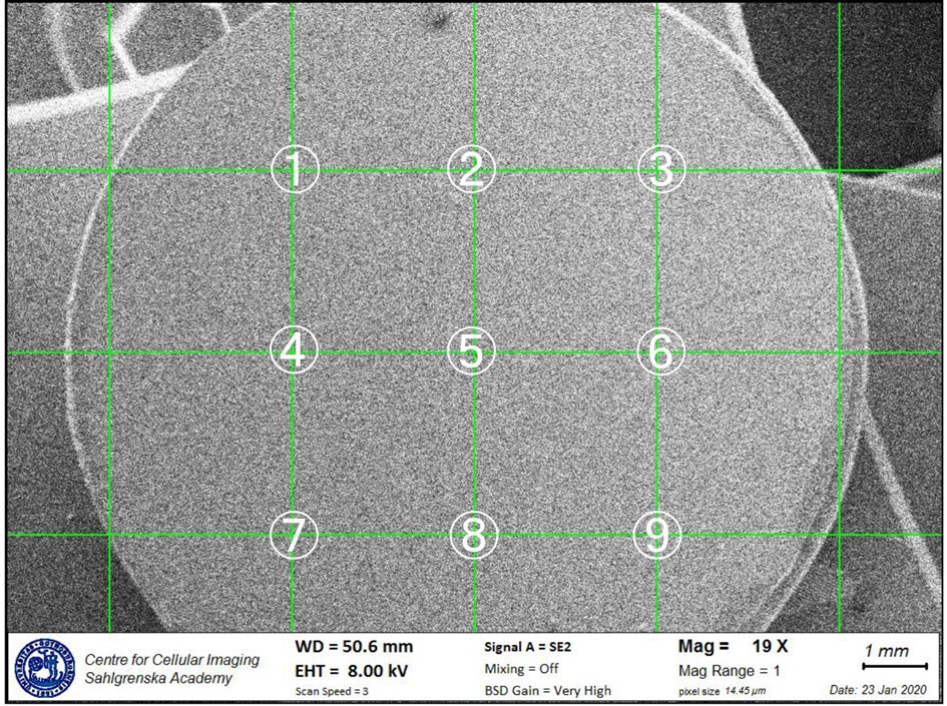
SEM image of a titanium disc with overlaid grid. Higher magnification images were obtained at the prespecified nine points.

For the image analysis, observation sites were selected by applying a grid with 9 cross points over the entire disc at a magnification of 19 × (Fig. 1). At each cross point, a micrograph was obtained. Coverage areas of cells, residual bacteria and material deposits were evaluated with an image analysis software (Fiji, National Institutes of Health, Bethesda, ML, USA) at a magnification of 2000 × using the SE2 detector.

### Immunofluorescence analyses

**Figure 2:**
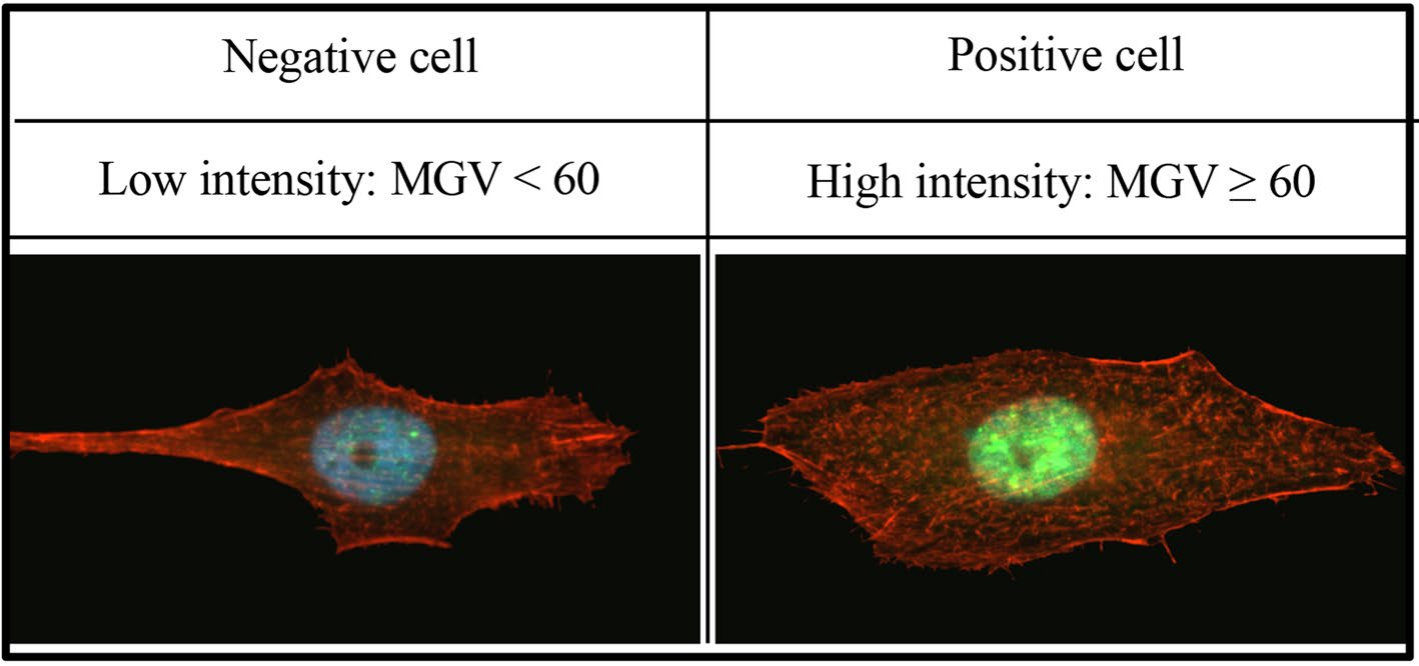
The mean grey value (MGV) based classification.

A fluorescent microscope (Leitz DM-RXA, Leica, Wetzlar, Germany) was used to evaluate the number of cells attached to the titanium surfaces as well as cell morphology and DNA damage. Six titanium discs (3 Ti(s) & 3 Ti(r)) from each of the control and the 4 different treatment groups were placed into a 24-wells plate immediately after surface decontamination and MG63-cells were seeded at a density of 1.0 × 10^4^ cells/cm^2^. After 24 h incubation in a humidified atmosphere of 95% air and 5% carbon dioxide at 37 °C, attached cells were fixed in 4% formaldehyde (VWR, Gothenburg, Sweden) for 10 min at room temperature and washed three times with PBS. The specimens were incubated in 0.1% Triton-X-100 (Sigma-Aldrich) in TBS (Sigma-Aldrich) for 15 min and subsequently with 1% bovine serum albumin (BSA, Sigma-Aldrich) for 60 min. After washing with TBS, cells were stained using fluorescent mouse anti-γH2AX monoclonal antibody (abcam 26350, 1:100, Cambridge, UK) followed by secondary antibodies conjugated with Alexa Fluor® 488 (Invitrogen, Fischer Scientific, Gothenburg, Sweden) and rhodamine phalloidin (abcam ab176757, 1:1000, Cambridge, UK). To visualize DNA content, cells were stained for 5 min in the dark using Hoechst (Invitrogen, Fischer Scientific, Gothenburg, Sweden) before being washed three times with PBS followed by rinsing twice with distilled water. Five pictures each at magnifications of 100 × and 400 × were obtained. Fluorescent microscopic images at 100 × magnification were used for counting of cells attached to titanium surfaces using the Cell Counter plugin system of the Fiji software. Individual cell areas were quantitatively assessed using fluorescent microscope images at 400 × magnification. Cells stained with γH2AX were graded by mean gray value (MGV). High intensity cells (MGV ≥ 60) were defined as γH2AX-positive cells (Fig. 2).

### Cytotoxicity of chemical agents

WST-8-based colorimetric assay (Cell Counting Kit-8, Dojindo, Kumamoto, Japan) was used to evaluate cell viability after exposure to the chemical agents (4 samples per group). MG63- and HGF-cells were seeded in 96-well plates at a concentration of 5000 cells/well. After 24 h incubation in a humidified atmosphere of 95% air and 5% carbon dioxide at 37 °C, the medium was removed and washed twice with PBS. Cells were incubated in 100 ml of fresh medium and 10 ml of either NaCl, AEW, CA or NAC. After 1 and 5 min of exposure, respectively, cells were washed twice with PBS and then cultured in 100 ml new medium and 10 ml reagent containing water-soluble tetrazolium salt (WST-8, Dojindo, Kumamoto, Japan) for 1 h incubation at 37 °C in a humid atmosphere under 5% CO_2_. Absorbance at 450 nm was measured using a microplate reader (MultiskanTM FC Microplate Photometer, Thermo Scientific, Waltham, Massachusetts, USA). The culture medium containing WST-8 without cells was used to set the background threshold, while culture medium containing WST-8 with cells was used as a control. Cell viability was determined through the following formula; Cell viability (%) = (A_sample_ − A_background_)/(A_Control_ − A_Background_) × 100.

Immunofluorescent staining with γH2AX was used to evaluate the amount of DNA damage after exposure to chemical agents. MG63- and HGF-cells were seeded on a glass slide (VWR international AB, Spånga, Sweden; diameter: 13 mm; thickness: 0.13 mm) in a 12-well plate at a concentration of 5,000 cells/well. After incubation for 24 h at 37 °C in a humidified incubator under 5% CO_2_, cells were gently washed twice with PBS and immersed in 500 ml of fresh medium and 50 ml of either NaCl, AEW, CA or NAC. After either 1 or 5 min of exposure, cells were washed twice with PBS and then fixed with 70% methanol at − 20 °C for 5 min. The cells were incubated with 0.1% Triton-X-100 in PBS and 3% bovine serum albumin (BSA) for 60 min. Cells were then incubated with primary antibody to γH2AX (Abcam ab62623, 1:50, Cambridge UK) at 4 °C overnight, rinsed three times with PBS and incubated with a secondary antibody conjugated with Alexa Fluor® 568 (Invitrogen, Fischer Scientific, Gothenburg, Sweden) for 2 h at room temperature. In addition, cells were stained for 5 min in the dark using Hoechst (Invitrogen, Fischer Scientific, Gothenburg, Sweden) in order to visualize DNA content. Five pictures from each specimen (3 samples per group) at a magnification of 200 × were obtained using a fluorescence microscope (Leitz DM-RXA, Leica, Wetzlar, Germany). Fluorescent microscopic images were used for counting of γH2AX-positive cell (MGV ≥ 60).

### Data analysis

Stata (Version 16, Stata Corporation, College Station, Texas) was used for data analysis. Multiple linear regression analysis was used to assess the effect of treatment method and surface category on (1) residual deposits, (2) cytocompatibility and (3) cytotoxicity. Interaction between independent parameters was explored. Statistical significance was set to *p* < 0.05.

## Results

**Figure 3:**
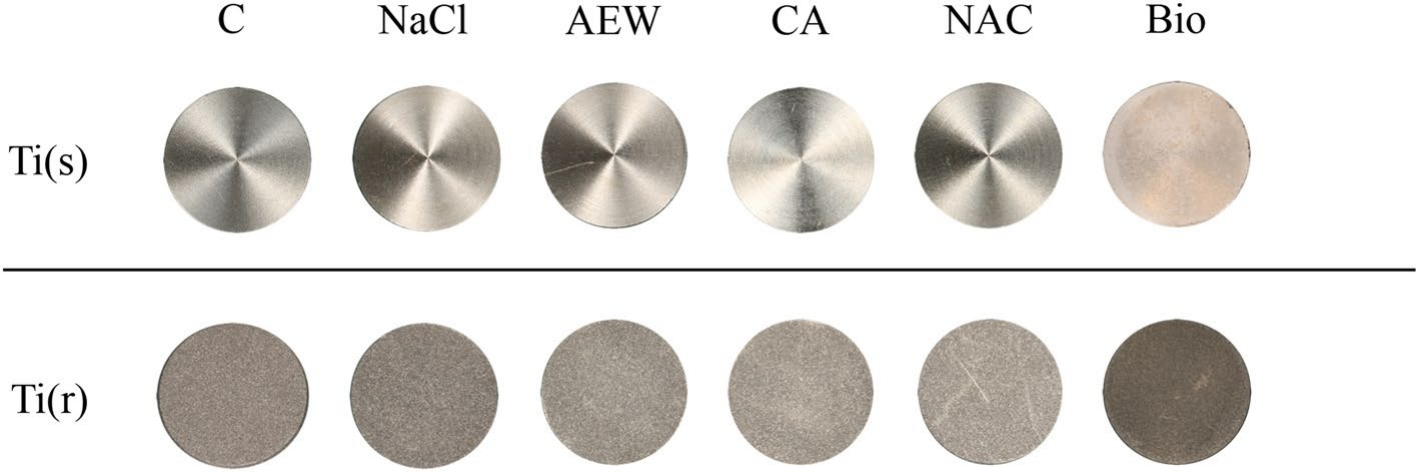
Photographic images of the two different titanium surfaces following application of four different decontamination methods (NaCl: gauze-soaked in saline, AEW: gauze-soaked in 0.1% alkaline electrized water, CA: gauze-soaked in citric acid, NAC: gauze-soaked in 10% *N*-acetyl-l-cysteine), control (C) and biofilm-contaminated discs (Bio).

Photographic images of discs following surface decontamination with four different chemical agents are shown in Fig. 3. Discs showed no visual surface alterations other than occasional scratch marks from dental tweezers. All methods seemed to effectively eliminate the *S. gordonii* biofilm as judged by ocular evaluation.

### SEM analysis: cytocompatibility and residual deposits

**Figure 4:**
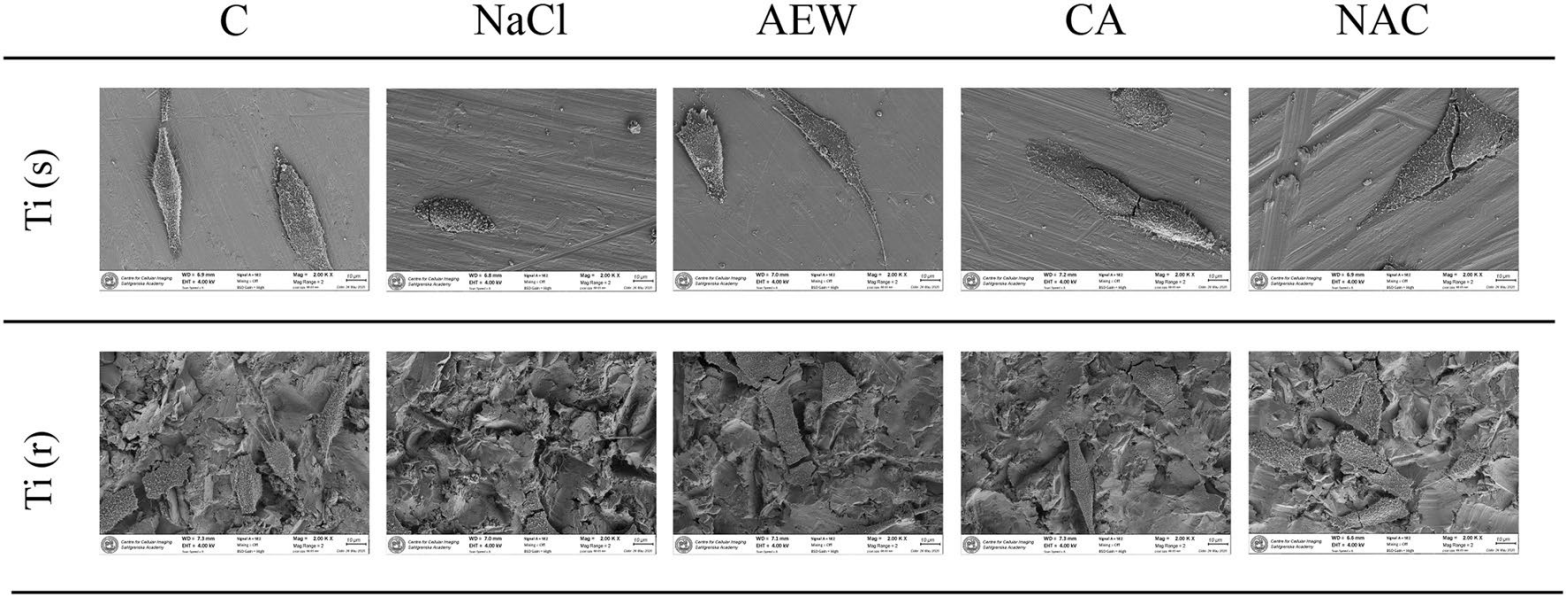
Representative SEM images of the two different titanium surfaces following application of four different decontamination methods and control discs.

SEM images of the two different types of titanium discs following application of the four different decontamination methods are shown in Fig. 4. Results of the analysis are presented in Table 1. Evaluating cytocompatibility, none of the treatment groups reached the level of pristine discs (C) in terms of mean %area of MG63-cells. Among treatment groups, AEW demonstrated the highest values and was superior to NaCl, as was NAC. These findings were consistent for non-modified (Ti(s)) and modified (Ti(r)) surface characteristics. In terms of residual bacteria, no significant differences between treatment groups were observed at Ti(s). At Ti(r), the mean %area of bacteria was significantly lower for all three chemical agents (AEW, NAC and CA) when compared to NaCl. In addition, AEW and CA showed lower amounts of residual bacteria than NAC. Gauze remnants on Ti(s) were rarely observed, while these were frequently detected on Ti(r) (Table 1).

**Table 1:** Mean %area of cells, bacteria and gauze deposit on titanium surfaces per SEM images at 2000 × magnification.

Group	Surface	Cells (%area)	vs. C (p value)	vs. NaCl (p value)	vs. AEW (p value)	vs. CA (p value)	Bacteria (%area)	vs. NaCl (p value)	vs. AEW (p value)	vs. CA (p value)	Gauze remnants (%area)	vs. NaCl (p value)	vs. AEW (p value)	vs. CA (p value)
C	Ti(s)	13.1 ± 1.3	–	–	–	–	–	–	–	–	–	–	–	–
	Ti(r)	11.3 ± 3.8	–	–	–	–	–	–	–	–	–	–	–	–
NaCl	Ti(s)	4.4 ± 0.9	0.000	–	–	–	0.11 ± 0.14 #	–	–	–	0.02 ± 0.03 #	–	–	–
	Ti(r)	3.6 ± 1.9	0.000	–	–	–	1.67 ± 0.65	–	–	–	0.55 ± 0.36	–	–	–
AEW	Ti(s)	9.6 ± 1.1	0.067	0.009	–	–	0.12 ± 0.06	0.969	–	–	0.01 ± 0.06 #	0.898	–	–
	Ti(r)	8.6 ± 3.3	0.145	0.012	–	–	0.08 ± 0.01	0.000	–	–	0.65 ± 0.16	0.418	–	–
CA	Ti(s)	6.3 ± 1.4	0.001	0.314	0.080	–	0.09 ± 0.09	0.904	0.874	–	0.02 ± 0.03 #	0.963	0.861	–
	Ti(r)	5.2 ± 3.1	0.003	0.386	0.078	–	0.15 ± 0.08	0.000	0.743	–	0.53 ± 0.14	0.916	0.362	–
NAC	Ti(s)	8.2 ± 1.7	0.013	0.048	0.449	0.296	0.04 ± 0.03 #	0.751	0.723	0.844	0.02 ± 0.03	0.991	0.889	0.972
	Ti(r)	8.1 ± 1.6	0.093	0.021	0.807	0.123	0.74 ± 0.41	0.001	0.010	0.020	0.23 ± 0.12	0.026	0.005	0.032

^#^indicates a significant difference (*p* < 0.05) between disc categories. All *p* values based on multiple regression analysis. Six discs (3 Ti(s) & 3 Ti(r)) per group, total n = 30 discs.

### Immunofluorescence analyses: cytocompatibility and DNA damages

**Figure 5:**
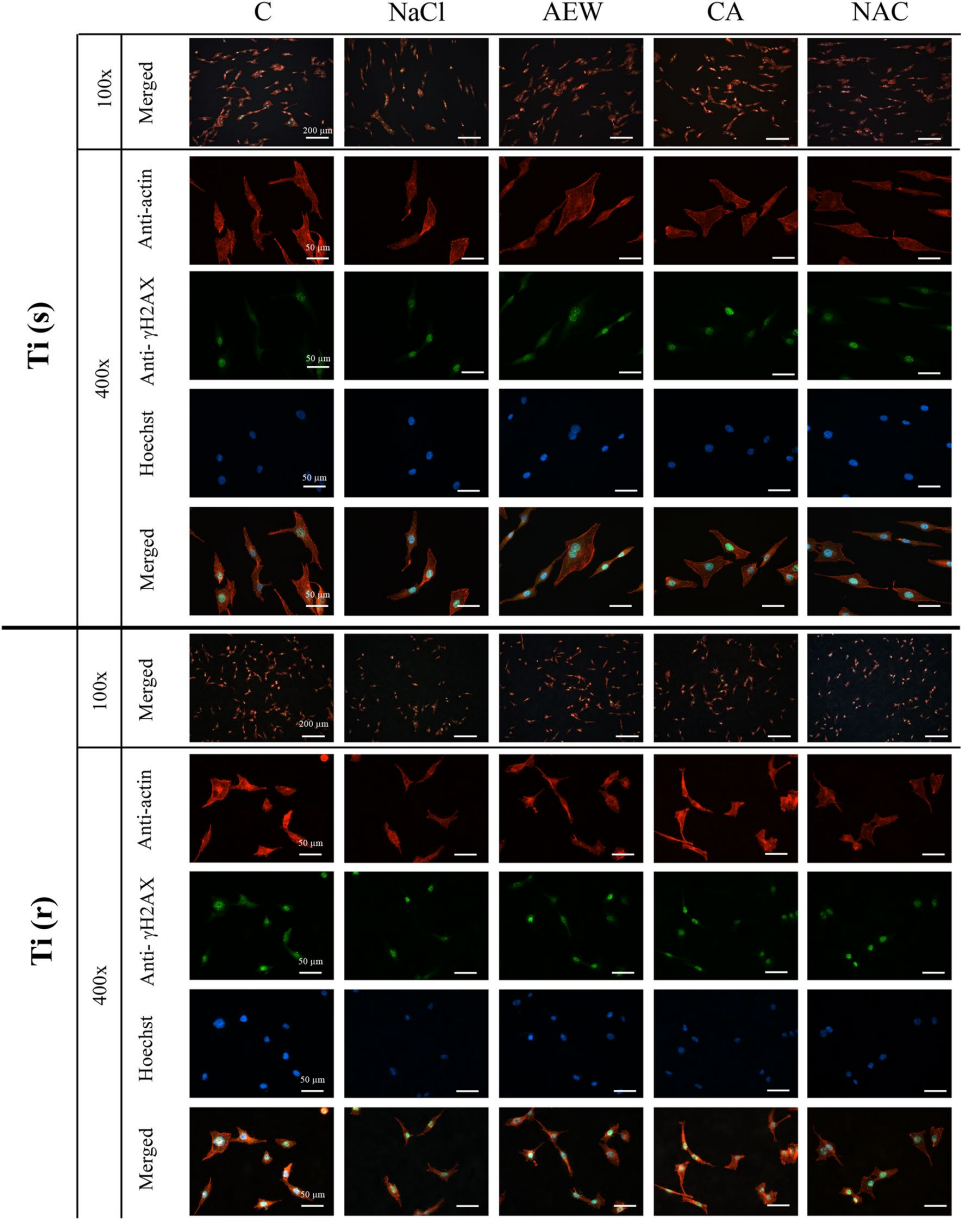
Representative fluorescent microscopic images of the two different titanium surfaces following application of four different decontamination methods and control discs.

**Figure 6:**
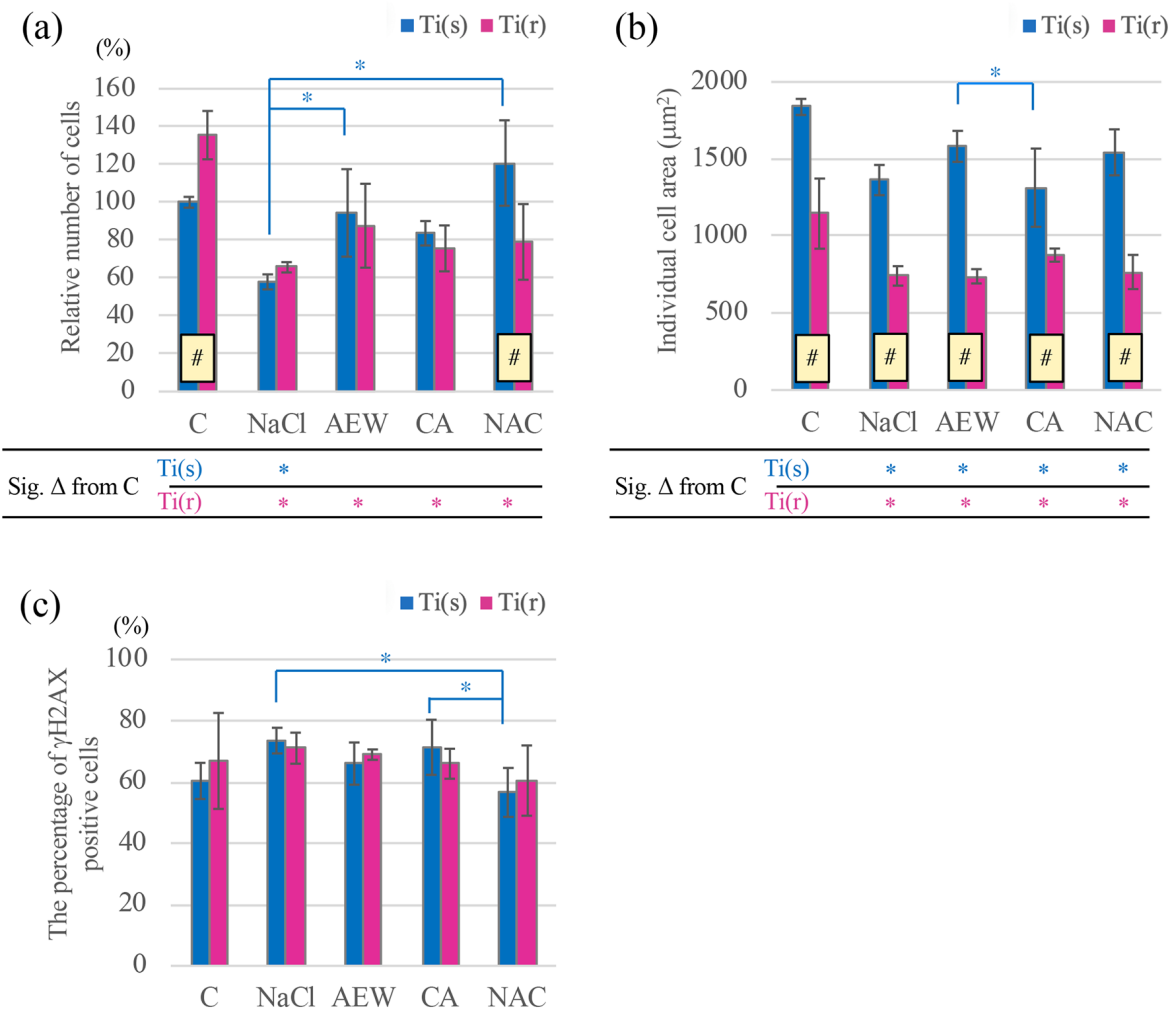
(**a**) Relative number of cells attached to control and to treated titanium discs. (**b**) Individual cell area (actin-stained) on titanium discs by group. (**c**) Percentage of γH2AX-positive cells on titanium discs by group. Mean values and ± SD; *indicates a significant difference (*p* < 0.05) between the (sub)groups and ^#^indicates a significant difference (*p* < 0.05) between disc categories. All *p* values based on multiple regression analysis. Six discs (3 Ti(s) and 3 Ti(r)) per group, total n = 30 discs.

Fluorescence images of cells and results of the image analysis are illustrated in Figs. 5 and 6. At Ti(s), the number of attached MG63-cells in AEW, CA and NAC were not significantly different from C (Fig. 6a), while NaCl showed inferior values. At Ti(r), all treatment groups were inferior to C with no differences between groups.

Cytomorphometric evaluation (Fig. 6b) showed that MG63-cells attached to surfaces treated with any of the chemical agents were significantly smaller than those on control surfaces for both Ti(s) and Ti(r). The actin-stained area on AEW-treated Ti(s) was significantly greater than that on CA-treated Ti(s), while no significant differences were found among treated Ti(r). In general, cells grown on Ti(s) were significantly larger than on Ti(r).

Higher percentages of γH2AX-positive MG63-cells were observed for NaCl- and CA-treated Ti(s) when compared to NAC (Fig. 6c). There were no significant differences between Ti(r) groups.

### Cytotoxicity of chemical agents

**Figure 7:**
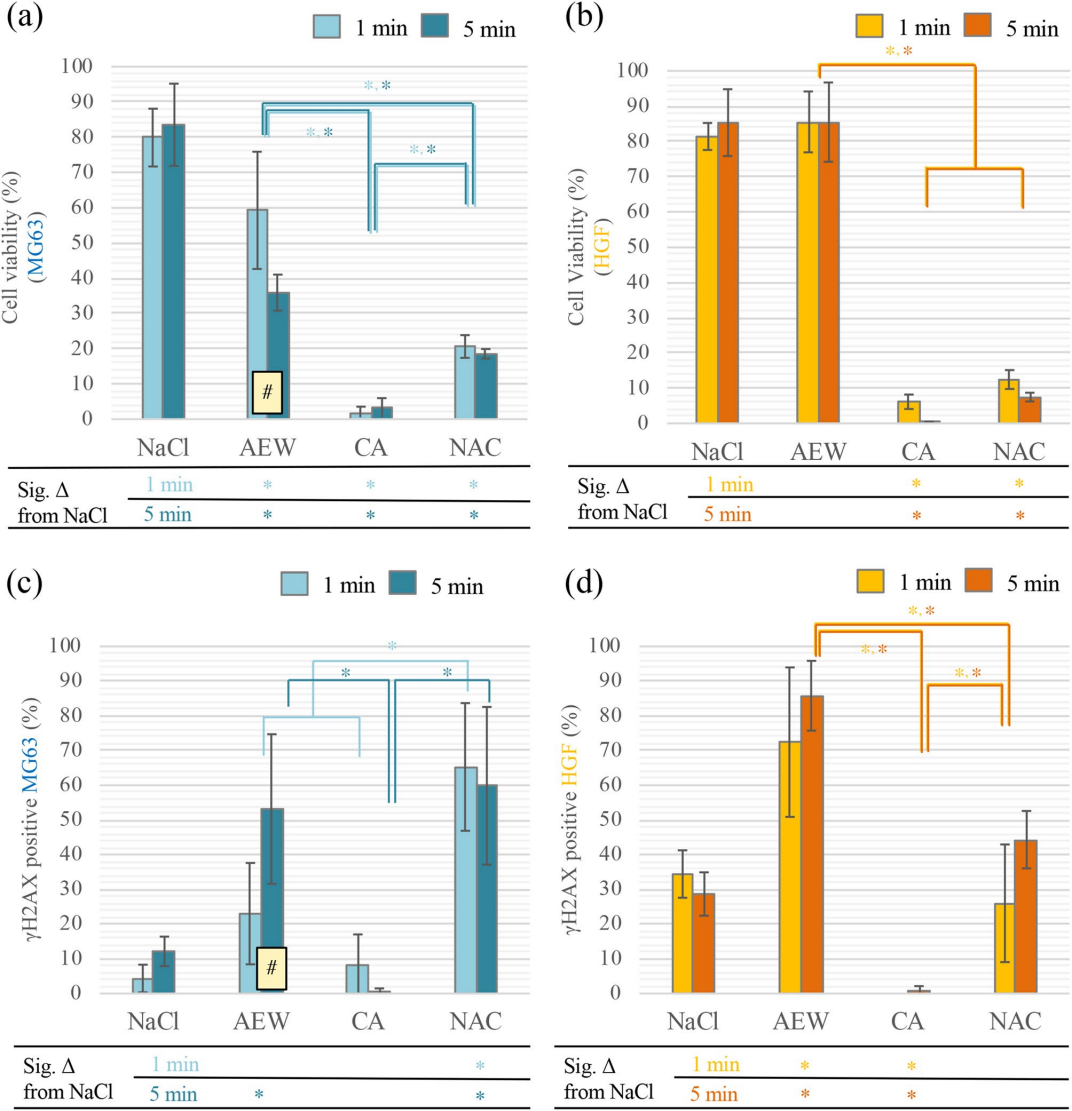
Cytotoxicity of chemical agents for osteoblast-like cells (MG63) and human gingival fibroblasts (HGF). (**a**) Cell viability of MG63 after 1 and 5 min. 4 samples per group evaluated by WST-8 assay. (**b**) Cell viability of HGF after 1 and 5 min. 4 samples per group evaluated by WST-8 assay. (**c**) Percentage of γH2AX-positive MG63-cells after 1 and 5 min. 3 samples per group evaluated by immunofluorescence. (d) Percentage of γH2AX-positive HGF-cells after 1 and 5 min. 3 samples per group evaluated by immunofluorescence. Mean values and ± SD; *indicates a significant difference (*p* < 0.05) between the (sub)groups and ^#^indicates a significant difference (*p* < 0.05) between time points. All *p* values based on multiple regression analysis.

Viability of MG63-cells after 1 and 5 min of NaCl exposure was significantly higher than for the other chemical agents. MG63-cell viability after AEW exposure at both time points was significantly higher when compared to exposure to CA and NAC. Time of exposure significantly affected cell viability of MG63-cells for AEW. The order of cytotoxicity for MG63-cells at both time points was NaCl < AEW < NAC < CA (Fig. 7a). HGF-cell viability after exposure to AEW was similar to NaCl. Likewise, HGF-cell viability after exposure to NAC was not different from CA. The order of cytotoxicity for HGF at both time points was NaCl = AEW < NAC = CA (Fig. 7b).

After 1 min, the percentage of γH2AX-positive MG63-cells exposed to NAC was significantly higher than for NaCl, AEW and CA. After 5 min, the percentage of γH2AX-positive MG63-cells exposed to AEW was significantly higher than for NaCl and CA. The order of DNA damage for MG63-cells at both time points was NaCl ≤ CA < AEW ≤ NAC (Fig. 7c). The percentages of γH2AX-positive HGF-cells after 1 and 5 min of exposure to AEW were significantly higher than for NaCl, CA and NAC. The percentages of γH2AX-positive MG63- and HGF-cells after CA exposure were considerably lower when compared to AEW and NAC. The order of DNA damage for HGF-cells at both time points was CA < NaCl = NAC < AEW (Fig. 7d).

## Discussion

The present *in-vitro* study demonstrated that chemo-mechanical cleaning of a modified (rough) implant surface (Ti(r)) with AEW and CA resulted in lower numbers of residual bacteria when compared to NaCl and NAC. There was no additional cleaning effect of any of the chemical agents at Ti(s). It was further observed that AEW and NAC were beneficial in restoring cytocompatibility. In addition, cytotoxicity of AEW was significantly lower when compared to CA and NAC.

The present observation that chemical agents were beneficial in biofilm removal is not in agreement with findings presented by Charalampakis et al.^29^. The authors evaluated the efficacy of saline, chlorhexidingluconate, delmopinol and a mixture of essential oils as adjuncts to mechanical debridement of contaminated titanium surfaces. None of the chemical agents demonstrated an additional benefit. It should be highlighted, however, that the two studies differed in terms of type of experimental biofilm as well as choice and application time of chemical agents. Disagreement may potentially also be explained by differences in quantities of liquids and mechanical pressure applied during decontamination. Neither volume nor pressure were standardized or assessed in the present study.

SEM analysis in the present study demonstrated that the mean %area of attached osteoblast-like cells following the use of AEW and NAC was greater when compared to NaCl, which suggests that the two active agents had a positive impact on cytocompatibility. A number of studies^15,30–32^ carried out similar evaluations. Three^30–32^ of these studies performed autoclave sterilization of the treated discs in advance to cell experiments making comparisons with the present findings difficult. Omitting autoclave sterilization, Ichioka et al.^15^ evaluated the effect of different solutions (NaCl, AEW and H_2_O_2_) as adjuncts to air-polishing on the restoration of surface chemical properties and cytocompatibility of experimentally contaminated (*S. gordonii*) titanium surfaces. It was demonstrated that AEW had the potential to remove organic impurities and rejuvenate surface chemical properties as well as to restore cytocompatibility. This observation is in line with findings of the present study showing superior results for AEW in terms of cytocompatibility.

According to the present findings, also NAC resulted in improved cytocompatibility when compared to saline. Enhanced alkaline phosphatase activity and mineralized matrix formation together with consistent upregulation of bone-related gene markers such as collagen I, osteopontin, and osteocalcin was reported following the use of NAC in vitro^33^. In addition, in an in vivo experiment^33^, NAC promoted bone healing in femoral critical size cortical bone defects in the rat model. These observations may serve as an explanation for the greater number of attached osteoblast-like cells recorded in our study.

It should be noted that the present data derived from an *in-vitro* experiment prohibiting clinical inferences. It is, for instance, not fully understood, whether the artificially produced biofilm adequately mimics the clinical situation in terms of adherence of bacteria to the underlying titanium surface.

Results of the WST-8 assay suggested that both NaCl and AEW exhibited high cell viability. Sipahi et al.^34^ examined cytotoxicity of electrolyzed water using a MTT assay. After an exposure of 24 h, no cytotoxicity was observed, which is in agreement with results of the present study. It should be noted, however, that the mean% of γH2AX-positive osteoblast-like and fibroblast cells for AEW were significantly higher than for NaCl. The DNA damage induced by AEW may be explained by the presence of HClO and ^–^OCl. Both are strong oxidizing agents and result in the formation of reactive oxygen species, such as superoxide, hydroxyl radicals and single oxygen^16,35^, which are all well-recognized mediators of DNA damage^36^. The discrepancy between the high cell viability concomitant with high levels of DNA damage following AEW treatment should be noted. While the expression of γH2AX represents an early cellular response to DNA double-strand breaks, it also indicates DNA repair mechanisms^37,38^.

Within the limitations of the present study, the combination of mechanical and chemical cleaning was effective in removing bacterial biofilm from non-modified and modified titanium surfaces and in restoring cytocompatibility. In particular the use of AEW was found to benefit biofilm removal and post-cleaning cytocompatibility, while demonstrating low levels of cytotoxicity.

## Data Availability

The dataset generated and analyzed in the current study is available from the corresponding author upon reasonable request.
